# Temporal Dynamics of Reward Dysfunction in Psychiatric Disorders: Memory-Derived Value, Incentive Salience, Hedonic Impact, and Value Updating

**DOI:** 10.1016/j.bpsgos.2026.100786

**Published:** 2026-07-04

**Authors:** Feng Zou, Xiaochen Yang, Congcong Liu, Kun Li, Meng Zhang

**Affiliations:** a From the Department of Psychology, Xinxiang Medical University, Xinxiang, China; b Mental Illness and Cognitive Neuroscience Key Laboratory of Xinxiang, Xinxiang Medical University, Xinxiang, China; c School of Psychology, Northwest Normal University, Lanzhou, China; d Shandong Daizhuang Hospital, Jining, China; e Jining Key Laboratory of Neuromodulation, Jining, China

**Keywords:** Anhedonia, Computational psychiatry, Incentive salience, Reward memory, Reward processing, Transdiagnostic psychiatry

## Abstract

Reward processing unfolds across time: Prior experiences shape cue valuation and motivation, anticipated rewards bias action selection, reward encounters generate hedonic and evaluative signals, and outcomes update future value representations. Existing accounts have often examined these processes separately. Here, we propose a temporally organized and mechanistically constrained framework for reward dysfunction across psychiatric disorders. The revised framework distinguishes episodic reward memory, cue-outcome learning, stored value representations, model-based prospective simulation, model-free habit learning, incentive salience, anticipatory pleasure, hedonic impact, outcome evaluation, effort valuation, and value updating. We argue that psychiatric disorders are not characterized best by single broken links in a reward cycle but rather by partially overlapping profiles of altered coupling among these processes. Major depressive disorder may involve impaired positive value updating and reduced access to positive reward memories; schizophrenia may involve unstable value representations, impaired effort-cost integration, and reduced model-based use of reward information; bipolar disorder may involve state-dependent reward hypersensitivity and regulatory instability; and addiction may involve cue-triggered incentive salience, habitization, and negative reinforcement. We specify how these profiles can be tested using reinforcement learning, effort-based decision making, memory-guided choice, ecological momentary assessment, and longitudinal paradigms. This framework generates falsifiable predictions and mechanistically informed intervention hypotheses while avoiding one-to-one mappings between diagnoses, symptoms, and neural circuits.

## Plain Language Summary

Many psychiatric disorders involve reduced pleasure or motivation, but these symptoms may arise from different reward-processing mechanisms. In this review, we explain reward dysfunction as a process that unfolds over time: Past reward memories shape expectations, cues influence motivation, outcomes generate pleasure and feedback, and experiences update future value. We distinguish these components in depression, schizophrenia, bipolar disorder, and addiction and propose testable ways to study them in the laboratory and everyday life. This framework may guide more precise research and cautious intervention hypotheses.

Reward seeking is central to adaptive behavior, learning, social affiliation, and survival. In psychiatry, disturbances of motivation and pleasure are clinically important because anhedonia and amotivation cut across diagnostic categories and are associated with functional impairment. Contemporary work has decomposed reward processing into partially dissociable processes, including reward responsiveness, learning, valuation, effort allocation, and action selection. The Research Domain Criteria initiative similarly locates reward responsiveness, reward learning, and reward valuation within the positive valence systems domain (1, 2, 3).

The idea that prior experience shapes future reward pursuit is not new. Reward-memory interactions have been extensively reviewed in the adaptive memory literature, and model-based reinforcement learning accounts already formalize how learned state representations guide value-based choice (4, 5, 6). Likewise, the distinction between incentive salience, or wanting, and hedonic impact, or liking, is central to modern reward neuroscience and addiction theory (7, 8, 9). Therefore, in the current article, we do not claim that memory, anticipation, and reward receipt have not been studied previously. Instead, we ask how these processes are temporally coupled, how the coupling can be operationalized, and how different psychiatric phenotypes may reflect different vulnerability profiles within this temporal architecture.

The contribution of the revised framework is 3-fold. First, it replaces a unitary notion of reward memory with a differentiated taxonomy that includes episodic reward memory, learned cue-outcome associations, semantic or stored value representations, model-based prospective simulation, and model-free or habitual control. Second, it maps temporal transitions in reward processing onto candidate neural systems, computational variables, and observable behavioral readouts. Third, it shifts the clinical claim from a single weak-link model to a dominant vulnerability profile model. We focus on major depressive disorder (MDD), schizophrenia, bipolar disorder (BD), and addiction because these conditions all include motivational or hedonic symptoms but differ in the processes most likely to be disrupted. For example, recent transdiagnostic work has shown that anhedonia relates to reduced striatal reward anticipation in depression but not equivalently in schizophrenia or BD, highlighting both overlap and diagnostic heterogeneity (10).

Accordingly, the framework is intended as a heuristic and testable organization of the literature, not as a biomarker model or a fully specified mathematical theory. Its central claim is that psychiatric reward dysfunction is best understood as altered temporal coupling among memory-derived value representations, cue-triggered incentive salience, anticipated pleasure or expected value, hedonic impact and outcome evaluation, effort valuation, value updating, and model-based versus model-free control.

## Definitions and Boundary Conditions

In this section, we clarify the constructs used throughout the review. We use reward memory as an umbrella term rather than a unitary mechanism. It includes episodic representations of specific rewarding events, learned cue-outcome associations, semantic or stored value representations, and procedural or habitual action tendencies. These processes are related but not interchangeable: Episodic memory supports context-rich recollection and prospective simulation, cue-outcome learning supports Pavlovian prediction, stored value representations guide choice, and habits support efficient but less flexible action selection (4,6,11,12).

Anticipatory pleasure refers to the conscious expectation that a future event will be pleasurable. Incentive salience, or wanting, refers to the motivational pull attributed to a cue or action; it can be triggered by Pavlovian cues and may occur without explicit awareness or without a conscious expectation of pleasure. This distinction is especially important for addiction, where sensitized wanting can drive drug seeking even when liking is diminished, and for schizophrenia, where immediate affective experience may not translate into motivated behavior (7,8,13).

Hedonic impact, or liking, refers to immediate affective pleasure. Outcome evaluation is broader and includes the cognitive and neural processing of feedback, such as monetary wins or losses, reward positivity, feedback-related negativity, or striatal prediction-error responses. In human monetary tasks, feedback should not be equated with consummatory pleasure in the strong hedonic sense. Monetary feedback is a useful operational proxy for outcome evaluation, but it differs from primary, social, aesthetic, or drug rewards in sensory and motivational properties (3,14,15).

Reward valuation and effort valuation are also distinct. Reward valuation concerns expected or subjective value; effort valuation concerns whether expected reward justifies physical or cognitive costs. Finally, model-based control uses an internal representation of states and future outcomes, whereas model-free or habitual control relies more heavily on cached values or stimulus-response tendencies (4,16,17). These distinctions are necessary because disorders can show superficially similar anhedonia or amotivation while differing in memory accessibility, cue salience, effort-cost computation, learning rate, or habit strength.

The framework also has domain boundaries. Monetary, social, natural, and drug rewards partially share valuation and motivational circuitry but are not interchangeable. Drug rewards recruit pharmacological reinforcement and withdrawal processes; social rewards depend on interpersonal meaning and social prediction; monetary rewards are abstract and experimentally convenient; and natural rewards involve sensory, homeostatic, and contextual components. Therefore, the framework generalizes most safely at the level of temporal coupling and computational readouts, not at the level of a single neural substrate for all reward types.

## Neurobiological and Computational Architecture of Temporal Reward Coupling

Reward processing depends on distributed systems rather than one-to-one mappings between psychological constructs and brain regions. Ventral tegmental area dopamine neurons, the ventral and dorsal striatum, the orbitofrontal cortex (OFC), the ventromedial prefrontal cortex, the anterior cingulate cortex, the hippocampus, the amygdala, the lateral habenula, and neuromodulatory systems all contribute to reward-related behavior depending on task state, reward domain, and computational demand (18, 19, 20).

Dopamine is strongly implicated in reward prediction errors, incentive salience, and motivated action, but it is not simply a pleasure signal. Classic prediction-error theory and later refinements show that dopamine signals support learning and action invigoration while also reflecting diversity across dopamine neuron populations (21, 22, 23). Hedonic impact is more closely associated with opioid-related mechanisms in hedonic hotspots, whereas serotonin, norepinephrine, and aversive systems modulate reward seeking under uncertainty, punishment, anxiety, and threat (15,24,25). This is relevant for MDD and addiction because reward deficits are often coupled to negative affect, avoidance, withdrawal, and punishment sensitivity rather than existing as isolated positive-valence deficits (26,27).

The hippocampus should not be reduced to episodic encoding and retrieval. It can support prospective simulation, value-based deliberation, relational state maps, and abstract representations of prospective reward (11,12,28). The OFC can represent task states and latent structure for value-based choice, and the amygdala-OFC system helps encode state-dependent, outcome-specific reward memories that can guide future decisions (29,30). These systems provide the neural basis for memory-derived value and prospective choice but do not imply that any single region uniquely belongs to a single temporal phase.

**Figure 1:**
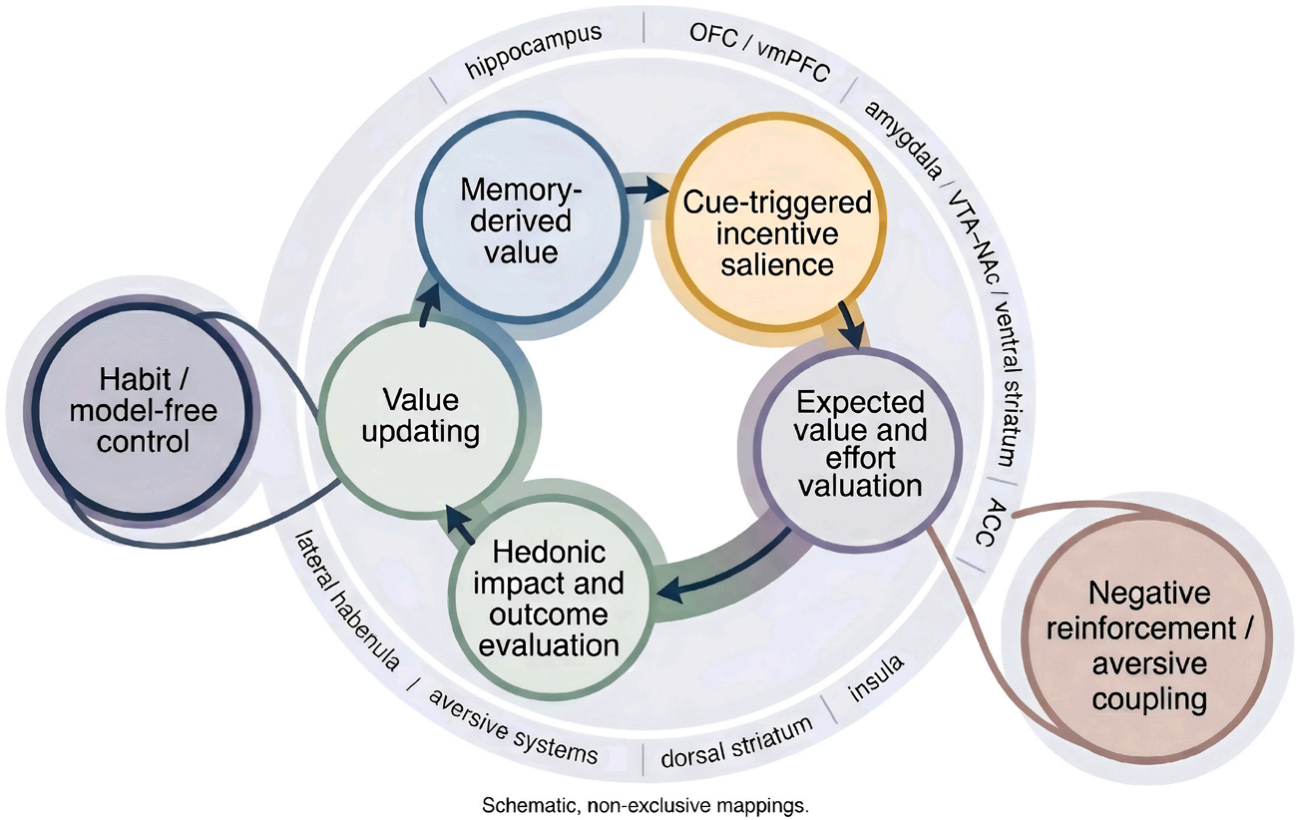
Temporal reward-coupling architecture. Reward processing is organized as partially overlapping temporal components rather than isolated, one-to-one modules. The schematic highlights memory-derived value, cue-triggered incentive salience, expected value and effort valuation, hedonic impact and outcome evaluation, and value updating, together with habit/model-free control and negative reinforcement/aversive coupling as related influences. Distributed neural systems are shown only as approximate, nonexclusive contributors. The figure is heuristic and should not be interpreted as implying unique mappings between individual constructs, disorders, and brain regions. ACC, anterior cingulate cortex; NAc, nucleus accumbens; OFC, orbitofrontal cortex; vmPFC, ventromedial prefrontal cortex; VTA, ventral tegmental area.

To avoid overclaiming, we do not propose a fully specified mathematical model. Instead, Table 1 provides an operational mapping between temporal transitions, psychological processes, candidate neural systems, computational variables, and measurable readouts. Reinforcement learning, active inference–inspired, and drift-diffusion approaches can be used to estimate these variables, but in the current review, they are treated as tools for future testing rather than as already validated disease-specific biomarkers (4,5,31). To clarify the conceptual structure of the framework, we present a schematic architecture that organizes reward dysfunction as a temporally coupled process spanning memory-derived value, incentive salience, effort-related valuation, outcome evaluation, and updating while also acknowledging habit formation and negative reinforcement as related influences (Figure 1).

**Table 1:** Mechanistic and Computational Mapping of Temporal Reward Coupling

Temporal Transition	Psychological Process	Candidate Neural Systems	Computational Variable	Behavioral/Neural Readout
Memory→Cue Valuation	Retrieval of value-relevant prior experience, contextual state representation	Hippocampus, OFC, vmPFC, amygdala	Prior value, state representation, memory precision	Memory-guided choice, cue valuation, recall specificity
Cue→Motivation	Incentive salience, Pavlovian invigoration	VTA-NAc, amygdala-OFC, striatum	Cue salience, Pavlovian bias	Craving, approach, Pavlovian-instrumental transfer
Anticipation→Effort	Expected value integrated with physical/cognitive cost	ACC, striatum, vmPFC	Effort-cost sensitivity, expected value of control	EEfRT, grip-force tasks, cognitive effort tasks
Outcome→Evaluation	Feedback processing and affective/cognitive appraisal	Striatum, OFC, ACC, insula	RPE (delta), outcome valence	RewP/FRN, striatal RPE, subjective ratings
Outcome→Memory Update	Updating stored value and future priors	Hippocampus, OFC, striatum	Positive/negative learning rates (alpha+/alpha−), memory decay	Trial-to-trial updating, delayed source memory
Repetition→Habit	Shift from goal-directed to habitual control	Dorsal striatum; PFC-striatal circuits	Model-free weight, habit strength	Outcome devaluation, slips of action, perseveration

ACC, anterior cingulate cortex; EEfRT, Effort Expenditure for Rewards Task; FRN, feedback-related negativity; NAc, nucleus accumbens; OFC, orbitofrontal cortex; PFC, prefrontal cortex; RewP, reward positivity; RPE, reward prediction error; vmPFC, ventromedial PFC; VTA, ventral tegmental area.

## Transdiagnostic Profiles of Reward-Cycle Dysregulation

The goal of the following sections is not to assign each disorder to one temporal phase but rather to specify the processes most likely to be altered, the key boundary conditions that limit diagnostic generalization, and predictions that could distinguish the framework from existing accounts.

### MDD: Impaired Positive Value Updating and Memory-Guided Prospection

MDD is associated with prominent reward-processing abnormalities, but it should not be reduced to a single anticipatory deficit. Many studies have implicated reduced reward anticipation, blunted striatal responses, impaired reward learning, and reduced willingness to exert effort for reward (14,32, 33, 34). Daniels *et al.* (10) provide a useful transdiagnostic anchor by showing that anhedonia relates to reduced striatal reward anticipation in depression but not equivalently in schizophrenia or BD. At the same time, psychophysiological and meta-analytic findings indicate that feedback and consummatory abnormalities may also occur, especially in particular demographic or symptom subgroups (14,35).

A plausible temporal chain is that negative or overgeneral autobiographical memory bias reduces access to specific positive reward memories; impoverished positive memory–guided prospection weakens expected value; reduced expected value and increased effort-cost sensitivity decrease reward pursuit; and fewer positive experiences further reduce opportunities for positive updating. Dillon *et al.* (36, 37, 38) have shown that weak memory for positive material in depression can reflect blunted dopaminergic midbrain and medial temporal responses during encoding, and Dillon’s review explicitly frames positive memory deficits as a reward-memory problem rather than only a mood-congruent recall bias.

Computationally, this profile can be expressed as blunted positive reward prediction errors, reduced positive learning rates, or pessimistic belief updating. Korn *et al.* (39) reported reduced optimistic belief updating in depression, and Kumar *et al.* (40) found impaired reward prediction error encoding and reduced striatal-midbrain connectivity in unmedicated MDD. Ecological momentary assessment (EMA) further supports a daily-life reward loop in which activity engagement, positive affect, motivation, and subsequent engagement influence each other over time (41,42). Together, these findings support a coupling account in which positive memory accessibility, reward updating, and effort valuation interact rather than a model in which depression-related anhedonia reflects a single anticipatory or consummatory deficit.

These predictions should be interpreted in light of heterogeneity within MDD. Immediate pleasure is not necessarily intact across all patients or tasks, and anticipatory, effort-related, feedback-related, memory-related, and consummatory abnormalities may vary with age, sex, symptom profile, reward domain, and illness stage. A critical test of the framework is whether reduced effort expenditure is better predicted by impaired positive memory accessibility and blunted positive learning rates than by immediate hedonic ratings alone.

### Schizophrenia: Value Representation, Effort-Cost Integration, and Model-Based Use of Reward Information

Negative symptoms in schizophrenia, including avolition and anhedonia, have often been described as a dissociation between relatively preserved in-the-moment pleasure and impaired future motivation. This distinction remains useful, but the revised framework avoids the stronger claim that consummatory pleasure is globally intact. Laboratory meta-analytic work indicates that emotional experience may be less globally blunted than self-reported trait anhedonia, but recent neuroimaging meta-analysis shows reduced activation across reward anticipation, consumption, learning, and effort computation components in the schizophrenia spectrum (43,44).

The mechanistic problem is better framed as difficulty forming, maintaining, and deploying value representations for goal-directed action. Gold *et al.* (45) and Strauss *et al.* (13) emphasized impaired value representation and motivational impairment. Effort-cost work further shows that patients with schizophrenia often choose high-effort options less frequently when rewards are available, although mechanisms may include underestimated reward value, overestimated effort cost, cognitive control limitations, defeatist beliefs, medication effects, or failure of accurate values to invigorate behavior (16,46,47).

Therefore, several mechanisms may contribute: unstable internal goal-value representations, working memory limitations, reduced model-based planning, altered prefrontal-striatal connectivity, and impaired effort-cost integration. The behavioral phenotype can be described as a reduced ability to use prior reward experience and current value information to guide future effortful behavior. This formulation preserves the clinical intuition behind a liking-to-action disconnect while avoiding an unsupported binary of intact consumption versus impaired anticipation. Taken together, these mixed findings argue against a simple preserved-liking/impaired-wanting dichotomy and instead point to failures in maintaining and using value representations for delayed, effortful, goal-directed action.

In schizophrenia, diagnostic and mechanistic heterogeneity constrains any single-process account. Medication status, negative symptom subtype, cognitive impairment, social versus nonsocial reward, and task demands all influence findings. A critical test is whether immediate hedonic ratings predict delayed effort allocation less reliably in individual with schizophrenia than in control individuals, especially when value information must be maintained across a delay without external reminders. If external scaffolding of goal value improves effort allocation, this would support a value-maintenance or model-based–control interpretation rather than a primary hedonic deficit.

### BD: State-Dependent Reward Hypersensitivity and Regulatory Instability

BD requires a different formulation from both MDD and schizophrenia. The original term global gain control was too broad unless formally defined. In the revised framework, gain is used only descriptively to refer to state-dependent amplification or attenuation of reward sensitivity, cue salience, goal pursuit, and feedback-based regulation; it is not treated as a formally estimated computational parameter. The most defensible claim is that BD involves state-dependent reward hypersensitivity and regulatory instability, especially in relation to mania and hypomania (48,49).

The empirical basis for this profile comes from behavioral activation system (BAS) models, goal-attainment findings, reward-related life-event work, and neuroimaging evidence of altered striatal and frontostriatal reward responses. Elevated striatal activity during reward anticipation has been reported in bipolar samples, including work suggesting elevated ventral striatal responses in bipolar II disorder and across monetary and social rewards (50, 51, 52). Furthermore, longitudinal and course-related studies suggest that reward sensitivity may relate to manic symptoms or recurrence, although findings depend on sample, mood state, medication, and measurement strategy (48,53). Goal-attainment events provide an empirical anchor for linking reward sensitivity to manic symptom increases (54).

Therefore, within the temporal-coupling framework, BD is not a stable deficit in a single phase. In manic or hypomanic states, reward cues may acquire excessive motivational salience, goal pursuit may persist despite negative consequences, and feedback-based downregulation may be weakened. In depressive or euthymic states, reward responsivity may be blunted, normal, or expressed outside striatal anticipation measures, and transdiagnostic data suggest that anhedonia in BD does not map onto striatal anticipation in the same way as in MDD (10,55). This pattern links apparently divergent findings by treating reward sensitivity as a within-person, state-dependent coupling problem rather than as a fixed deficit or enhancement.

Because BD is intrinsically state dependent, the relevant prediction concerns within-person changes across manic, hypomanic, depressive, euthymic, and mixed states rather than a stable impairment in one reward phase. Cue-triggered approach, postreward persistence, and feedback-based downregulation should vary with mood state and BAS sensitivity.

### Addiction: Cue-Triggered Incentive Salience, Habitization, and Negative Reinforcement

Addiction illustrates how reward processing can become dominated by maladaptive cues, habits, and negative reinforcement. Drug-associated cues can acquire excessive incentive salience, triggering wanting and craving even when explicit expected pleasure or hedonic impact is reduced. This distinction is central to incentive-sensitization theory and directly motivates the framework’s separation of wanting from anticipatory pleasure and liking (7,8,56).

Repeated drug use also changes control of behavior. The transition from voluntary use to compulsive drug seeking has been described as a shift from goal-directed action to habit and compulsion, with increasing involvement of dorsal striatal systems and reduced prefrontal regulatory control (57, 58, 59, 60). This transition does not mean that memory disappears; rather, drug-related episodic, emotional, associative, and procedural memories become selectively weighted toward drug cues and contexts.

The revised framework also incorporates stages and negative valence. Early substance use may involve strong pharmacological reinforcement and positive incentive value. With repeated use, cue-triggered wanting, habitization, tolerance, withdrawal, stress, and opponent-process dynamics increasingly shape behavior, including experimental evidence for hedonic allostasis during escalating cocaine self-administration (61). Koob and Volkow’s neurocircuitry model emphasizes incentive salience, habit formation, and negative reinforcement, while the hyperkatifeia account explains how withdrawal-related negative affect provides a powerful source of motivation for compulsive use (26,27,62). These stage-related findings support an integrative account in which drug cues, habitual control, and negative affect become increasingly coupled over time.

**Figure 2:**
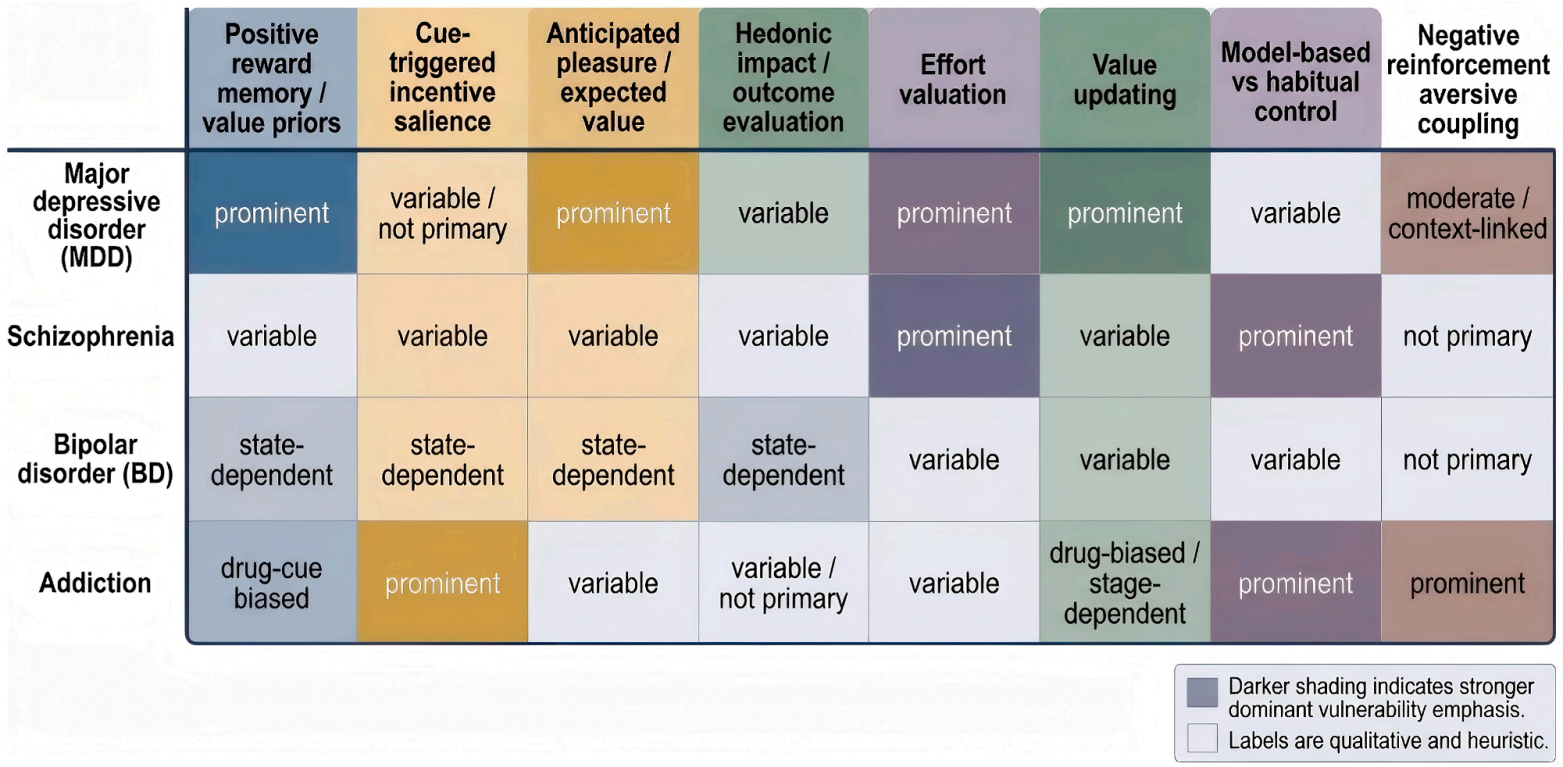
Dominant vulnerability profiles across temporal reward components. The revised framework characterizes psychiatric disorders as profiles of altered coupling across reward components rather than as single broken links in a reward cycle. The heatmap summarizes dominant vulnerabilities across positive reward memory/value priors, cue-triggered incentive salience, anticipated pleasure/expected value, hedonic impact/outcome evaluation, effort valuation, value updating, model-based vs. habitual control, and negative reinforcement/aversive coupling. Labels are intentionally qualitative and heuristic. The figure should not be interpreted as a biomarker map or as evidence of one-to-one disorder-mechanism mappings.

For addiction, the dominant temporal coupling is expected to vary by substance class, illness stage, abstinence or withdrawal status, environmental context, and comorbid affective symptoms. In later-stage or withdrawal-related contexts, cue-triggered wanting and negative reinforcement should predict drug seeking more strongly than explicit expected pleasure, whereas early-stage use may be more closely related to expected positive reinforcement. To summarize these disorder-related patterns while avoiding one-disorder/one-breakpoint assumptions, heuristic profiles of dominant vulnerability across temporal reward components are presented in Figure 2. Table 2 complements Figure 2 by summarizing each disorder profile together with key boundary conditions, candidate computational variables, and falsifiable predictions.

**Table 2:** Disorder Profiles, Key Boundary Conditions, Candidate Variables, and Falsifiable Predictions

Disorder	Dominant Vulnerability Profile	Key Boundary Conditions	Candidate Variables/Readouts	Falsifiable Prediction
MDD	Impaired positive value updating and reduced positive memory–guided prospection	Heterogeneous across symptom profile, reward domain, and illness stage; not limited to anticipation	Positive RPE, alpha+, positive memory accessibility, effort-cost sensitivity, EMA reward loop strength	Positive memory accessibility and alpha+ should predict effort expenditure better than immediate liking alone.
Schizophrenia	Unstable value representation, effort-cost integration, and reduced model-based use of reward information	Consumption is not globally intact; findings vary by task, symptoms, medication, and reward domain	Value maintenance, working memory, effort-cost sensitivity, model-based control weight	External goal-value scaffolding should improve liking-to-effort transfer if value maintenance is the limiting mechanism.
BD	State-dependent reward hypersensitivity and regulatory instability	Profiles vary across manic, hypomanic, euthymic, depressive, and mixed states	BAS sensitivity, cue salience, temporal discounting, feedback-based downregulation	Cue-triggered approach and postreward persistence should vary with mood state and BAS sensitivity.
Addiction	Cue-triggered incentive salience, habitization, and negative reinforcement	Varies by substance, stage, abstinence or withdrawal status, environment, and comorbidity	Pavlovian bias, craving, model-free weight, withdrawal-related negative affect, temporal discounting	Later-stage drug seeking should be predicted more strongly by cue-triggered wanting and negative reinforcement than by explicit expected pleasure.

BAS, behavioral activation system; BD, bipolar disorder; EMA, ecologic momentary assessment; MDD, major depressive disorder; RPE, reward prediction error.

## Testing the Temporal-Coupling Framework

The framework should be evaluated by tasks that distinguish temporal coupling among constructs rather than merely by tasks that isolate single reward phases. Conventional tasks such as the monetary incentive delay task are valuable and often allow prediction error–based trial-to-trial modeling. The limitation is not that such tasks necessarily reset the internal state on every trial; rather, they usually do not jointly separate episodic memory retrieval, cue-triggered wanting, anticipated pleasure, effort valuation, hedonic impact, outcome evaluation, delayed updating, and later memory accessibility within one design.

A minimal experimental test should combine reward-memory or cue-outcome encoding; later retrieval or cue valuation; separate measurement of anticipated pleasure and cue-triggered wanting; effort-based choice or action vigor; reward receipt and outcome evaluation; trial-to-trial value updating; delayed memory testing; and computational fitting of learning rates, prediction errors, effort-cost sensitivity, temporal discounting, habit weight, or memory decay. Such designs would allow investigators to ask whether, for example, MDD is better explained by reduced positive updating, schizophrenia by impaired value maintenance, BD by state-dependent salience amplification, or addiction by cue-triggered wanting and habit strength.

The framework is falsifiable. It would be weakened if disorder-related phenotypes were fully explained by a single global reward-responsivity factor without separable contributions from memory accessibility, cue salience, effort-cost integration, outcome evaluation, value updating, or habit control. It would also be weakened if the predicted coupling patterns failed to generalize across independent laboratory, EMA, and longitudinal datasets.

**Figure 3:**
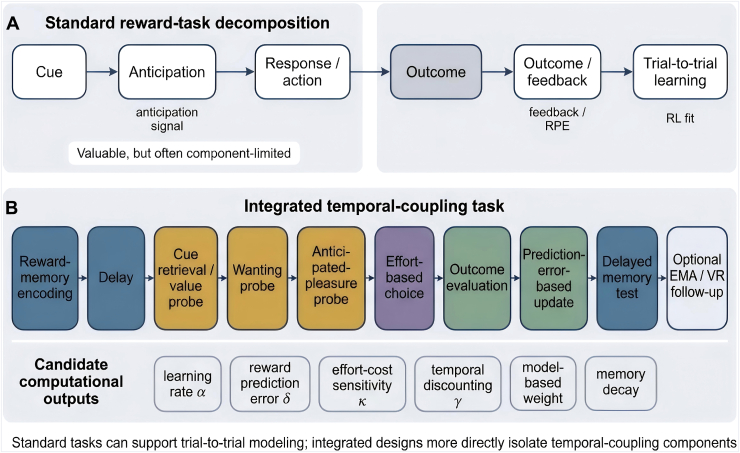
Testing temporal reward coupling. Standard reward tasks can be highly informative and may support trial-to-trial computational modeling, but they often do not jointly isolate all of the components emphasized by the temporal-coupling framework. Panel **(A)** shows a conventional phase-segmented reward-task decomposition. Panel **(B)** shows an integrated temporal-coupling design that includes reward-memory encoding, retrieval/value probing, separate wanting and anticipated-pleasure assessments, effort-based choice, outcome evaluation, updating, delayed memory testing, and optional ecological validation through ecological momentary assessment (EMA) or virtual reality (VR). The figure also illustrates candidate computational outputs that can be estimated from such designs. RL, reinforcement learning; RPE, reward prediction error.

EMA can provide ecological validation by testing whether laboratory-estimated parameters predict real-world sequences such as reward opportunity, positive affect, effort, social engagement, and subsequent motivation. This approach is especially relevant to depression, where daily-life reward-loop functioning can be assessed repeatedly over time (41,42). Virtual reality may be useful when experiments require controlled but naturalistic spatial or social contexts, including memory-guided navigation, social reward anticipation, and cue-triggered craving. To illustrate how these processes can be tested empirically without dismissing the value of existing paradigms, Figure 3 contrasts a conventional reward-task decomposition with an integrated temporal-coupling design. To further clarify what the current framework adds beyond existing approaches, in Table 3, it is compared with several influential models and the testable distinctions emphasized in this review are highlighted.

**Table 3:** How the Temporal-Coupling Framework Differs From Existing Models

Existing Framework	Core Emphasis	Strength	What the Current Framework Adds/Testable Distinction
RDoC Positive Valence Systems	Reward responsiveness, reward learning, reward valuation	Strong construct organization across diagnoses	Adds tests of temporal coupling among memory accessibility, cue salience, effort valuation, outcome evaluation, and value updating
Incentive-Sensitization Theory	Cue-triggered wanting in addiction	Explains craving despite reduced liking	Extends the wanting/liking distinction cautiously beyond addiction while preserving addiction-specific evidence; tests cue-triggered wanting against explicit anticipated pleasure
Computational Anhedonia/RL Models	Prediction error, learning rate, effort cost	Strong formalization of latent mechanisms	Links latent computational variables to memory-derived value, delayed updating, effort valuation, and EMA-validated reward loops
Memory-Guided Decision Making	Hippocampal/OFC state and value representations	Strong account of memory-choice interaction	Extends hippocampal/OFC memory-choice mechanisms to transdiagnostic psychopathology and mechanistically informed intervention hypotheses
Current Framework	Temporal coupling among memory, cue salience, effort, outcome, updating, and control	Generates cross-disorder testable profiles	Avoids 1-diagnosis/1-deficit mappings and specifies disorder-profile predictions that can be falsified

EMA, ecologic momentary assessment; OFC, orbitofrontal cortex; RDoC, Research Domain Criteria; RL, reinforcement learning.

## Mechanistically Informed Intervention Hypotheses

The clinical implications of this framework should be treated as hypotheses rather than treatment recommendations. Behavioral activation for MDD is already an evidence-based intervention; the framework helps reinterpret it mechanistically as a way to increase reward engagement, positive affect, and opportunities for positive updating, but it is not a novel prediction of the model (63).

For schizophrenia, the most defensible clinical implication is not that mindfulness can bridge liking and wanting. A more precise hypothesis is that interventions may need to scaffold goal-value representations, reduce defeatist beliefs, support effort allocation, and make links between positive experience and future action explicit (16,46,47,64). For BD, intervention hypotheses should focus on mood-state monitoring, rhythm stabilization, relapse prevention, and reward-focused psychoeducation rather than unsupported neuromodulatory stabilization of a global gain parameter. For addiction, cue exposure, extinction, reconsolidation, contingency management, and goal-directed retraining are plausible targets, but their relevance depends on substance type and illness stage (26,58).

Neuromodulatory approaches, including cerebellar or prefrontal stimulation, should be presented as preliminary experimental approaches unless supported by convergent clinical evidence. Therefore, in Table 4, candidate mechanisms are separated from evidentiary status, and conservative wording is used.

**Table 4:** Mechanistically Informed Intervention Hypotheses and Evidentiary Status

Target Process	Candidate Approach	Evidence Status	Recommended Wording
Reduced Reward Engagement and Positive Updating in MDD	BA, activity scheduling, positive memory work	Established treatment evidence for BA; mechanism can be reframed	Consistent with the framework
Effort/Value Integration and Value Maintenance in Schizophrenia	Goal-value scaffolding, cognitive-motivational interventions, effort training, defeatist-belief targeting	Emerging and heterogeneous	Candidate hypothesis
State-Dependent Reward Dysregulation in BD	Rhythm stabilization, relapse prevention, reward-focused psychoeducation, mood-state monitoring	Indirect support from clinical and reward-sensitivity models	May help regulate reward pursuit
Cue-Triggered Craving, Habit, and Negative Reinforcement in Addiction	Cue exposure/extinction, reconsolidation approaches, contingency management, goal-directed retraining	Mechanistically plausible; evidence varies by substance and stage	Testable intervention target
Circuit-Level Modulation of Reward Coupling	Prefrontal/cerebellar or other neuromodulation	Preliminary; not established as transdiagnostic regulator	Early-stage experimental approach

BA, behavioral activation; BD, bipolar disorder; MDD, major depressive disorder.

## Conclusions

Reward dysfunction is temporally structured. Prior experience shapes cue valuation and motivated action; anticipated rewards influence effort and affective context; reward receipt generates hedonic and evaluative signals; and outcomes update future value representations. The revised framework does not claim that each psychiatric disorder has a single broken link. Instead, it organizes MDD, schizophrenia, BD, and addiction as partially overlapping profiles of altered coupling among reward memory, incentive salience, anticipated pleasure, hedonic impact, outcome evaluation, effort valuation, value updating, and goal-directed versus habitual control.

This framework is useful only if it improves testability. The central next step is to design tasks and longitudinal studies that can estimate memory accessibility, cue-triggered wanting, effort-cost sensitivity, learning rate, temporal discounting, habit strength, and daily-life reward-loop functioning within the same individuals. If these variables distinguish clinical profiles better than global anhedonia scores, the framework will have heuristic value. If they do not, the framework should be revised. Therefore, the main promise is not immediate precision treatment but rather a more cautious and mechanistically explicit route for connecting symptoms, neural systems, computational variables, and real-world reward behavior.
